# Modulation of *Bifidobacterium* by HD5 during weaning is associated with high abundance in later life

**DOI:** 10.1038/s43856-025-00977-6

**Published:** 2025-07-01

**Authors:** Yu Shimizu, Yuki Yokoi, Shuya Ohira, Hirohisa Izumi, Satomi Kawakami, Miu Ihara, Fuka Tabata, Yasuhiro Takeda, Takashi Kimura, Koshi Nakamura, Akiko Tamakoshi, Tokiyoshi Ayabe, Kiminori Nakamura

**Affiliations:** 1 Innate Immunity Laboratory, Faculty of Advanced Life Science, Hokkaido University, Hokkaido, Japan; 2 R&D Division, Morinaga Milk Industry Co., Ltd., Kanagawa, Japan; 3 Global Research Center for Food and Medical Innovation, Institute for the Promotion of Business-Regional Collaboration, Hokkaido University, Hokkaido, Japan; 4 Graduate School of Life Science, Hokkaido University, Hokkaido, Japan; 5 Creative Research Institution, Hokkaido University, Hokkaido, Japan; 6 Department of Public Health, Faculty and Graduate School of Medicine, Hokkaido University, Hokkaido, Japan; 7 Department of Public Health and Epidemiology, Graduate School of Medicine, University of the Ryukyus, Okinawa, Japan

**Keywords:** Microbiome, Microbiota, Innate immunity

## Abstract

**Background:**

*Bifidobacterium* colonization of the intestine is believed to have beneficial effects on our health from infancy throughout life. However, how particular members of the genus *Bifidobacterium* colonize the neonatal intestine and whether early-life bifidobacterial colonization affects establishment of *Bifidobacterium*-rich microbiota in later life remain unanswered. α-Defensin secreted from small intestinal Paneth cells elicits selective bactericidal activities that efficiently kill pathogens while hardly affecting commensals including *Bifidobacterium* in vitro, thus contributing to intestinal microbiota regulation.

**Methods:**

One hundred forty-eight fecal samples were serially obtained from 33 children from postnatal 3–5 days to 3 years old, conducting a longitudinal cohort study of mothers and children living in Iwamizawa city, Hokkaido, Japan (SMILE Iwamizawa study). Microbiota composition and secretory level of α-defensin, human defensin 5 (HD5), were assessed to investigate the relationship between HD5 and *Bifidobacterium* colonization.

**Results:**

We show that HD5 is associated with colonization of *Bifidobacterium* in early life from pre-weaning to weaning periods. Furthermore, high relative abundance of *Bifidobacterium* in the weaning period, which positively correlates with HD5 secretion, is associated with the establishment of *Bifidobacterium*-rich microbiota at 3 years old, when the intestinal microbiota matures.

**Conclusions:**

This study suggests the importance of the weaning period in establishing long-lasting homeostasis interwoven with the host innate immunity and *Bifidobacterium* in the intestinal microbiota.

## Plain language summary

*Bifidobacterium* is a beneficial bacterium that lives in the intestine and supports our health from infancy to adulthood. However, it is not fully understood how these bacteria first colonize the babies’ intestine, or whether this early colonization affects the intestinal environment in later life. We followed 33 children from 3–5 days to 3 years old and collected fecal samples to study their intestinal bacteria. We also measured an antimicrobial peptide called HD5, which is known to control the intestinal bacteria. We found that HD5 modulates the colonization of *Bifidobacterium*, and that a high level of these bacteria during weaning may help establish a healthy intestinal environment that lasts. These findings suggest that supporting intestinal health during weaning benefit long-term well-being.

## Abstract (web summary)

Shimizu et al. investigate whether early-life human defensin 5 (HD5), a host innate immunity factor, influences *Bifidobacterium* colonization in infant gut and modulates microbiota development in later life. HD5-associated colonization during weaning contributes to the long-term establishment of a beneficial microbiota.

## Introduction

The concept of Developmental Origins of Health and Disease (DOHaD) that environmental factors during the first 1000 days of life, from embryonic period to the first 2 years of life, affect future risks of various diseases such as obesity, diabetes, cardiovascular diseases, and allergy has been widely recognized^1–3^. The intestinal microbiota is a complex ecosystem that influences a wide range of host physiology^4,5^, and early-life intestinal microbiota is considered as a factor that influences health and disease in later life. The immune and nervous systems rapidly develop from neonatal to early childhood, and the intestinal microbiota plays an important role^6,7^. It has been known that exposure to environmental factors that disrupt the intestinal microbiota such as cesarean section, malnutrition, and antibiotics administration in early life is associated with future increased risks of diseases such as allergic diseases, obesity, and developmental disorders^8–13^, suggesting that the establishment of a healthy intestinal microbiota in early life is crucial to promote lifelong health.

Several members of *Bifidobacterium* increase rapidly in the human intestinal tract from the first few days after birth and become one of the dominant bacteria in the early-life intestine^14^. It has been reported that *Bifidobacterium* in early life promotes maturation of host immune system^15–17^ and tissue development, such as the brain and intestine^18,19^. Furthermore, the establishment of *Bifidobacterium*-rich intestinal microbiota in early life is associated with the reduced risk of various diseases such as atopy, asthma, obesity, and developmental disorders in later life^20–23^, suggesting that *Bifidobacterium* in early life contribute to future health by promoting appropriate physiological development of host. Although decreasing upon weaning, *Bifidobacterium* is also one of the major genera in the adult intestine^24^. Furthermore, several epidemiologic studies suggest that the establishment of a *Bifidobacterium*-rich microbiota not limited in early life contributes to health from adolescence to old age^24–27^. The intestinal microbiota of children develops rapidly from immediately after birth and matures with forming the structure similar to that of adults by ~3 years old^28,29^. In addition, some strains of *Bifidobacterium* detected in feces of children with in the first year of life are also detected in their childhood (6–11 years old), showing long-term colonization of *Bifidobacterium* to the intestine^30,31^. Thus, it is supposed that higher relative abundance of *Bifidobacterium* in the early-life microbiota supports the establishment of *Bifidobacterium*-rich microbiota in later life via inheriting its population, and further contributes to lifelong health. However, whether *Bifidobacterium* in the early-life microbiota affects the relative abundance of *Bifidobacterium* in the intestinal microbiota at maturity remains unknown because there have been no longitudinal comprehensive analyses for the effect of *Bifidobacterium* from the neonatal to infancy on the future intestinal microbiota.

Paneth cells, a lineage of small intestinal epithelial cells, contribute to the regulation of the intestinal microbiota^32–34^ by secreting antimicrobial peptides, α-defensins termed cryptdin (Crp)s in mice and human defensin (HD) 5 and 6 in humans^35–39^, into the intestinal lumen in response to bacteria^40,41^ and food components^42^. Recently, it has been reported that abnormal Crp secretion is involved in the pathological progression of mouse models of several diseases, including Crohn’s disease and depression via inducing imbalance of the intestinal microbiota, dysbiosis^43–46^. Decrease of α-defensin expression in Paneth cells is also reported in patients with Crohn’s disease^47^. Furthermore, decreased levels of HD5 secretion relate to disturbance of the intestinal microbiota by aging^48^ and short sleep^49^ in humans. Expression of α-defensins in Paneth cells is already detected in the fetal small intestine of both mice and humans^50,51^. In mice, Paneth cell α-defensin is also known to increase rapidly during birth to weaning (21 days after birth)^52^. Infants suffered with necrotizing enterocolitis (NEC) are reported to have fewer number of Paneth cells^53^. In addition, Crps possess selective bactericidal activities in vitro, which strongly kill both pathogenic and opportunistic bacteria whereas shows no or minimal bactericidal activities against commensal bacteria, including *Bifidobacterium bifidum* (*B. bifidum*) and *Bifidobacterium breve* (*B. breve*)^54^. Therefore, it is suggested that maturation of α-defensin secretion in early life plays an important role in the establishment of the intestinal microbiota in later life via promoting the colonization of *Bifidobacterium* in the early-life intestine. However, the maturation process of α-defensin secretion in early life and the effects of α-defensin on *Bifidobacterium* in the human intestinal microbiota remain unknown.

In this study, we analyzed the transition of the relative abundance of *Bifidobacterium* and HD5 secretion during the early-life period and further evaluate its effects in the establishment of *Bifidobacterium*-rich microbiota in later life by conducting the Survey of Mothers, Infants, and Children’s Lives and Environments in Iwamizawa (SMILE Iwamizawa), the ongoing longitudinal cohort study targeting mothers and children living in Iwamizawa city, Hokkaido, Japan^55^. Here we show that the intestinal microbiota of children shows a mature composition similar to that of their mothers by 3 years old. Additionally, the relative abundance of *Bifidobacterium* at weaning and at 3 years old shows a positive correlation. Furthermore, the secretory level of HD5 positively correlates with the relative abundance of *Bifidobacterium* throughout the infancy, especially at the weaning. These results indicate that *Bifidobacterium* colonization during the weaning period, which is modulated by HD5 secretion, contributes to the development of *Bifidobacterium*-rich microbiota in later life, indicating the importance of the weaning period in establishing long-term health through the intestinal microbiota.

## Methods

### Study design and population

This study was conducted as a part of the SMILE Iwamizawa, an ongoing longitudinal cohort study in Iwamizawa, Hokkaido, Japan. The cohort study of mothers and children from pregnancy to the postpartum period began in January 2017 and will continue until 2027 to explore factors that influence children’s growth and development^55^. Pregnant women living in Iwamizawa are recruited when the municipal government issues the Maternal and Child Health Handbook and are enrolled in the cohort if informed consent is obtained. As a part of the survey, fecal samples are collected from children at 3–5 days old (d), 1 month old (m), 4–5 m, 8–9 m, 1.5 years old (y), 3 y, 5 y, and school-age. Fecal samples are also obtained from mothers at 24 weeks of gestation and when their children reach 3–5 d, 1 m, and 4–5 m. A small part of each fecal sample is collected by brush-type collection kits containing guanidine thiocyanate (Techno Suruga Laboratory Co., Ltd., Shizuoka, Japan) for the intestinal microbiota analysis. The remaining fecal samples are stored at −80 °C until use and subjected to a series of analyses, including HD5 quantification. In addition, anthropological data of the children measured at the time of regular check-ups are provided by the mothers at each time point.

At the time of this study (December 2022), 257 mother-child dyads were enrolled in the cohort, and pairs of data on fecal HD5 and the intestinal microbiota at 3 y were obtained from 33 children, who composed the most advanced group in the cohort. From these 33 children, pairs of HD5 and intestinal microbiota data were obtained from 152 fecal samples in total (*n* = 22, 21, 21, 27, 28, and 33 at 3–5 d, 1 m, 4–5 m, 8–9 m, 1.5 y, and 3 y, respectively). Also, 28 pairs of data were obtained from their mother at 4–5 m. From these 180 samples, 4 samples were excluded due to insufficient data quality and quantity of bacterial 16S rRNA gene sequencing (all samples were obtained from children at 1 m. Further details can be found in the Methods section under Bacterial 16S rRNA gene-based taxonomic analysis). Finally, 176 samples were analyzed in this study. This study was conducted in accordance with the guidelines of the Declaration of Helsinki and all procedures involving human subjects were approved by the ethics committees of the Graduate School of Medicine at Hokkaido University and the Morinaga Milk Industry Co., Ltd (approval number: 16-039 and 16005-144). Written informed consent was obtained from all mothers, and research consent from children was obtained on the basis of the consent signatures of their mothers.

### Bacterial 16S ribosomal RNA gene sequencing

Total genomic DNA was extracted from the fecal samples by bead-beating and purified for intestinal microbiota analysis by using the automated DNA Extraction system Gene Prep Star PI-480α (Kurabo Industries, Ltd, Osaka, Japan)^56^. The V3-V4 region of the bacterial 16S ribosomal RNA (rRNA) gene was subsequently amplified by using TaKaRa Ex Taq HS Kit (TaKaRa Bio Inc., Shiga, Japan) with the primer sets Tru357F (5′-CGCTCTTCCGATCTCTGTACGGRAGGCAGCAG-3′) and Tru806R (5′-CGCTCTTCCGATCTGACGGACTACHVGGGTWTCTAAT-3′) and sequenced via the pair-end method on a MiSeq instrument (SY-410-1003, Illumina Inc., Hayward, CA)^57^.

### Bacterial 16S rRNA gene-based taxonomic analysis

Paired-end fastq files obtained from MiSeq were demultiplexed and analyzed by the QIIME2 platform (version 2022.8)^58^. Quality-filtering, denoising, pair-end merging, and chimera removal of sequences were conducted via the qiime dada2 denoise-paired command of the DADA2 plugin^59^ with the following parameters: --p-trim-left-f 23; --p-trim-left-r 23; --p-trunc-len-f 270; --p-trunc-len-r 210; --p-max-ee-f 2; --p-max-ee-r 2. After this step, samples for which more than 2000 reads could not be obtained were excluded (4 samples obtained from children at 1 m) because sequence data quality and quantity were considered insufficient for the analysis. All remaining reads were rarefied to 2000 per sample. The phylogenic tree was subsequently constructed via FastTree2^60^ after alignment with MAFFT^61^ using the qiime phylogeny align-to-tree-mafft-fasttree command. The taxonomy of each feature at the phylum and genus level was assigned by the qiime feature-classifier classify-sklearn command using a naïve Bayes classifier trained on the GreenGenes2 2022.10 database^62^. For the species-level analysis of *Bifidobacterium*, taxonomic assignments were conducted by aligning each 16S rRNA V3-V4 sequence to the RefSeq database of 16S rRNA gene sequences released by NCBI (https://ftp.ncbi.nlm.nih.gov/refseq/TargetedLoci/Bacteria/; updated at 2023.4.15) using the qiime feature-classifier classify-consensus-blast command with the parameter --p-perc-identity 0.99. The α-diversity (observed features) and β-diversity (weighted UniFrac distance) were calculated by the qiime diversity alpha-rarefaction and qiime diversity core-metrics-phylogenetic commands, respectively. The statistical significance of β-diversity was determined via the permutational multivariant analysis of variance (PERMANOVA) test by using the qiime diversity beta-group-significance command. Additionally, a linear discriminant analysis effect size (LefSe) test^63^ was conducted by the web-based Galaxy platform (https://huttenhower.sph.harvard.edu/galaxy/).

### Calculation of body mass index (BMI) and BMI percentile

BMI and BMI percentile based on the sex-matched Japanese growth curve at 3 y were calculated from weight and height by using a Microsoft Excel-based tool for growth evaluation provided by the Japanese Society for Pediatric Endocrinology (http://jspe.umin.jp/medical/chart_dl.html). The participants were subsequently divided into LowBMI (*n* = 7; BMI percentile ≤ 33.3rd), MidBMI (*n* = 11; 33.4–66.7th percentile), and HighBMI (*n* = 14; >66.7th percentile) groups.

### Quantification of fecal HD5 by sandwich ELISA

Fecal samples were lyophilized and pulverized to powder using a bead-beating homogenizer (PV1001, Yasui Kikai, Corp., Osaka, Japan). Ten milligrams of fecal powder was suspended in 100 μL of PBS (−), vortexed at 4 °C overnight, and centrifuged at 15,000 × *g* for 30 min at 4 °C. Then, the supernatants were subjected to the previously developed sandwich ELISA system using in-house produced mouse anti-HD5 monoclonal antibodies (clones 12-1 and 39E-7)^48^.

### Preparation of the HD5 peptide

Chemically synthesized HD5 peptide (Thermo Fisher Scientific, Waltham, MA) was dissolved in water containing 3 mM reduced-form glutathione, 0.3 mM oxidized-form glutathione, and 8 M urea. Then, the solution was adjusted to pH 8.4 by adding 0.25 M NaHCO_3_ and air-oxidation was conducted by gently mixing at 4 °C overnight. Oxidized-form HD5 with three intramolecular disulfide bonds was purified by reverse-phase high-performance liquid chromatography by a C18 column (06526-21, Nacalai Tesque, Inc., Kyoto, Japan) in 0.1% trifluoroacetic acid with a 0-40% acetonitrile gradient developed over 50 min at a flow rate of 1 mL/min^48,54^.

### Bactericidal assay of HD5

*Bifidobacterium breve* (*B. breve*) JCM 1192, *Bifidobacterium longum* (*B. longum*) ATCC15707, *Lactobacillus casei* (*L. casei*) ATCC393, *Escherichia coli* (*E. coli*) ML35 ATCC 43827, *Staphylococcus aureus* (*S. aureus*) ATCC27212, and *Bacteroides fragilis* (*B. fragilis*) JCM11019 were used for the analysis. Each bacterium was cultured in the following media: *B. breve* and *B. longum*, reinforced clostridial medium (CM0149, Oxoid Ltd., Hampshire, UK); *L. casei*, de Man, Rogosa, Sharpe broth (CM0359, Oxoid Ltd.); *E. coli* and *S. aureus*, tryptic soy broth (211825, Becton, Dickinson and Company); *B. fragilis*, Gifu anaerobic medium (05422, Shimadzu Diagnostics Company, Tokyo, Japan). *E. coli* and *S. aureus* were cultured under aerobic condition at 37 °C with shaking at 180 rpm, and other bacteria were cultured under static, anaerobic conditions using the Anaero Pack system (A-02, Mitsubishi Gas Chemical Co., Inc., Tokyo, Japan) at 37 °C. Bacterial growth was monitored by measuring the optical density at 600 nm until reaching the exponential phase and then, the bacterial cultures were centrifuged at 3000 × *g* for 5 min at 4 °C. After the supernatant was removed, the bacteria were resuspended in 0.2 mM PIPES buffer. Ten microliters of bacterial suspension was mixed with an equal volume of oxidized-form HD5 dissolved in 0.2 mM PIPES buffer to the final concentrations of 0, 0.01, 0.03, 0.11, 0.33, and 1 μM, and then reacted for 1 h at 37 °C. After the reaction, the mixtures were spread on agar plates and incubated at 37 °C. The bacterial survival rates at each concentration of oxidized-form HD5 were calculated from the number of surviving colonies relative to peptide-unexposed controls (0 μM).

### Statistical analysis

All the statistical analyses were conducted with GraphPad Prism version. 9.0 software (GraphPad Software Inc., San Diego, CA). For all correlation analyses, Spearman’s rank correlation coefficient test was used. For group comparisons, the Mann-Whitney’s U test and one-way analysis of variance (ANOVA) followed by Dunnett’s test or Tukey’s multiple comparison test, were used depending on the situation. For all statistical tests, *p* < 0.05 was considered statistically significant.

### Reporting summary

Further information on research design is available in the Nature Portfolio Reporting Summary linked to this article.

## Results

### The intestinal microbiota of children matures by 3 y

**Fig. 1:**
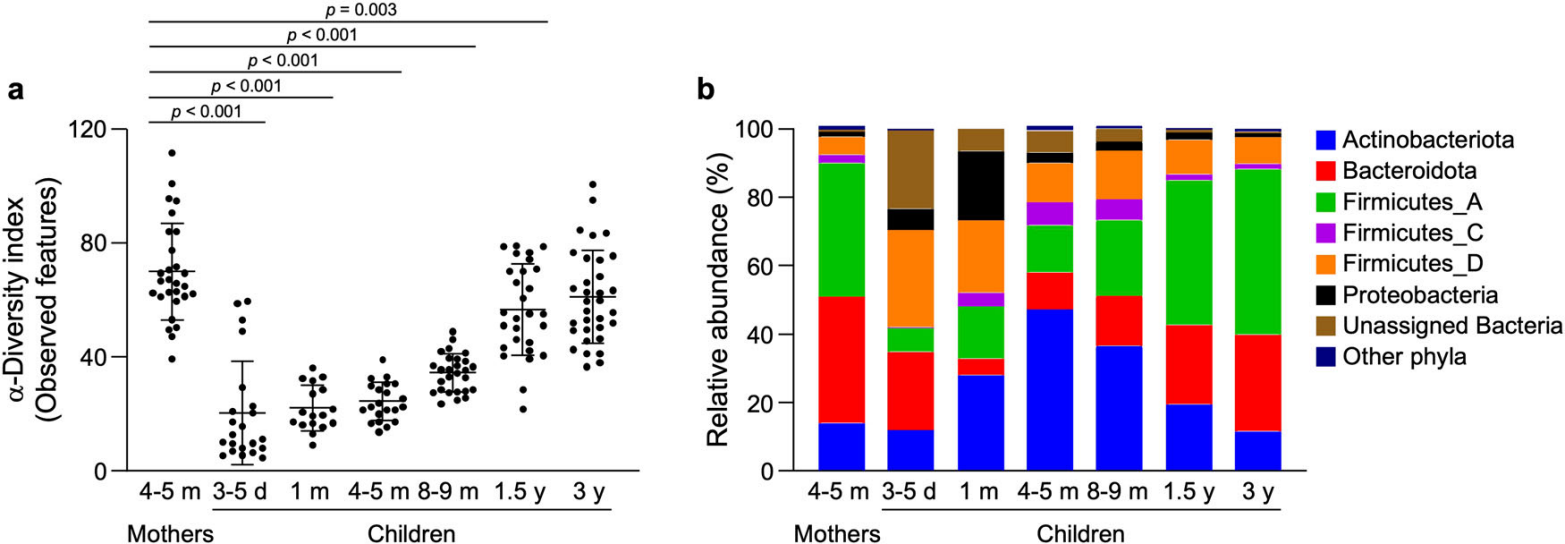
The intestinal microbiota of children maturates along with the development. **a** Comparison of an α-diversity index, observed features between mothers at 4–5 m (*n* = 28) and children at each time point (*n* = 22 at 3–5 d; *n* = 17 at 1 m; *n* = 21 at 4–5 m; *n* = 27 at 8–9 m; *n* = 28 at 1.5 y; *n* = 33 at 3 y). **b** Stacked bar chart of relative abundance of the intestinal microbiota at the phylum level. The error bars represent the means ± SD. Statistical significance between mothers and children at each age was evaluated by one-way ANOVA followed by Dunnett’s multiple comparison test in (**a**). *p* < 0.05 was considered statistically significant.

Among the participants in the SMILE Iwamizawa study, 33 children having the data on both fecal HD5 concentrations and bacterial 16S rRNA gene sequence with sufficient quality and quantity at 3 y were enrolled in this study. Then, total of 176 fecal samples obtained longitudinally from the children at 3–5 d (*n* = 22), 1 m (*n* = 17), 4–5 m (*n* = 21), 8–9 m (*n* = 27), 1.5 y (*n* = 28), and 3 y (*n* = 33) and their mothers at 4–5 m (*n* = 28) were included in the analysis (Table 1, Supplementary Tables 1 and 2). First, the maturation process of the children’s intestinal microbiota was analyzed. The α-diversity of the children’s microbiota increased along with their growth and reached the same level as that of mothers at 3 y (Fig. 1a). At the phylum level, Firmicutes- and Bacteroidota-dominant microbiota similar to those of their mothers were observed in the children after 1.5 y (Fig. 1b), indicating that the intestinal microbiota of the children matured by 3 y.

**Table 1:** Anthropological data of children who participated in this study

	3–5 d	1 m	4–5 m	8–9 m	1.5 y	3 y
Number of participants (male/female)	22 (16/6)	17 (13/4)	21 (16/5)	27 (18/9)	28 (19/9)	33 (23/10)
Weight (kg, mean ± SD)	3.1 ± 0.3	4.4 ± 0.6	7.4 ± 0.9	8.4 ± 1.1	10.6 ± 1.2	13.8 ± 2.0
Height (cm, mean ± SD)	49.0 ± 1.7	54.5 ± 2.1	64.6 ± 2.8	69.5 ± 3.0	80.6 ± 3.0	93.1 ± 4.0^a^
Body mass index [(BMI), kg/m^2^, mean ± SD]	13.1 ± 0.9	14.9 ± 2.0	17.6 ± 1.8	17.4 ± 1.6	16.3 ± 1.1	15.9 ± 1.3^a^
Head circumference [(HC), cm, mean ± SD]	33.5 ± 1.1	36.8 ± 1.1	42.1 ± 1.3	43.9 ± 1.5	46.6 ± 1.3	49.5 ± 1.3
Chest circumference [(CC), cm, mean ± SD]	31.9 ± 1.4	36.1 ± 1.3	43.4 ± 2.1	45.1 ± 2.4	47.2 ± 2.0	Not measured

^a^One participant denied the measurement of height.

### High relative abundance of *Bifidobacterium* at the weaning period is associated with the establishment of *Bifidobacterium*-rich microbiota at 3 y

**Fig. 2:**
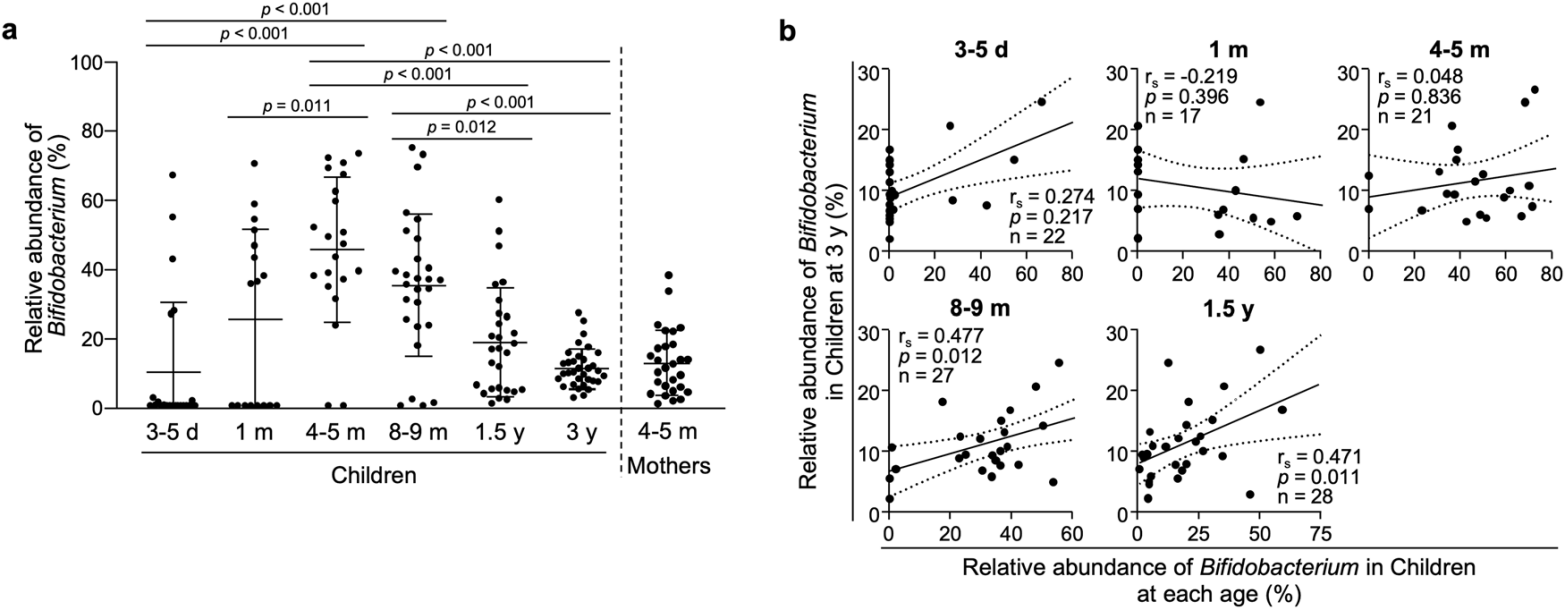
High relative abundance of *Bifidobacterium* in children at the weaning period is associated with the formation of a *Bifidobacterium*-rich microbiota at 3 y at the genus level. **a** Transition of the relative abundance of the *Bifidobacterium* genus in children. **b** Correlation analysis of the relative abundance of the *Bifidobacterium* genus between children at 3 y and those of each age. The dashed lines in (**b**) represent the 95% confidence interval range. Statistical significance among children at each age was evaluated by one-way ANOVA followed by Tukey’s multiple comparison test in (**a**) and Spearman’s rank correlation coefficient test in (**b**).

**Fig. 3:**
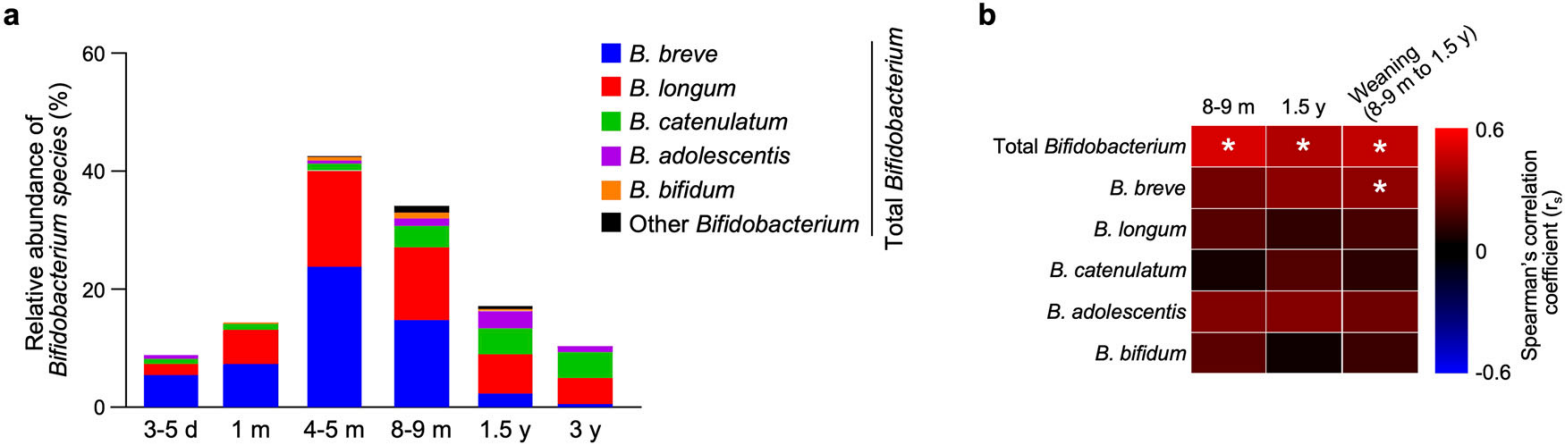
High relative abundance of *Bifidobacterium* in children at the weaning period is associated with the formation of *Bifidobacterium*-rich microbiota at 3 y at the species level. **a** Stacked bar chart of the relative abundance of *Bifidobacterium species* in children at each age. **b** Correlation matrix between the relative abundance of total *Bifidobacterium* in children at 3 y and each *Bifidobacterium species* in children at 8–9 m, 1.5 y, and the weaning period (combined data set of 8–9 m and 1.5 y). Statistical significance was evaluated by Spearman’s rank correlation coefficient test in (**b**). In (**b**), * in each cell indicates statistically significant correlation (*p* < 0.05).

Next, the relationships between the relative abundance of *Bifidobacterium* in the intestinal microbiota at 3 y, when mature, and that at each timepoint from newborn to infancy were analyzed (Supplementary Fig. 1). The relative abundance of the *Bifidobacterium* genus in the children increased from 3–5 d to 4–5 m, then decreased from 8–9 m and reached approximately the same level as that of the mothers at 3 y (Fig. 2a). Furthermore, the relative abundance of the *Bifidobacterium* genus at 3 y was positively correlated with that at 8–9 m and 1.5 y, whereas no significant correlation was detected before 4–5 m (Fig. 2b). In the species-level analysis, five taxa of *Bifidobacterium* species with average relative abundance greater than 0.1% were assigned (Fig. 3a, Supplementary Fig. 2). The relative abundance of total *Bifidobacterium*, the sum of these 5 species, at 3 y was positively correlated with that at 8–9 m and 1.5 y, whereas no significant correlations were detected between the relative abundance of total *Bifidobacterium* at 3 y and that of individual *Bifidobacterium* species at each age (Fig. 3b, Supplementary Fig. 3). Given the guidelines from the Ministry of Health, Labor and Welfare in Japan and the World Health Organization, which recommend weaning between 5–6 m to 1.5 y and between 6 m to 2 y, respectively^64,65^, we integrated the data from children at 8–9 m and 1.5 y as the weaning period which may more appropriately reflect the children’s developmental process and further analysis was conducted. As a result, *B. breve* at the weaning period was positively correlated with total *Bifidobacterium* at 3 y (Fig. 3b, Supplementary Fig. 3). In addition, a positive interspecies correlation was observed between *B. breve* at the weaning period and *B. catenulatum* at 3 y (Supplementary Fig. 4). These results indicate that a high relative abundance of *Bifidobacterium* at the weaning period is involved in the establishment of a *Bifidobacterium*-rich microbiota at 3 y, which represents a future timepoint.

### A high relative abundance of *Bifidobacterium* at 3 y is associated with a low BMI and the intestinal microbiota formation characterized by low relative abundance of *Parabacteroides*

**Fig. 4:**
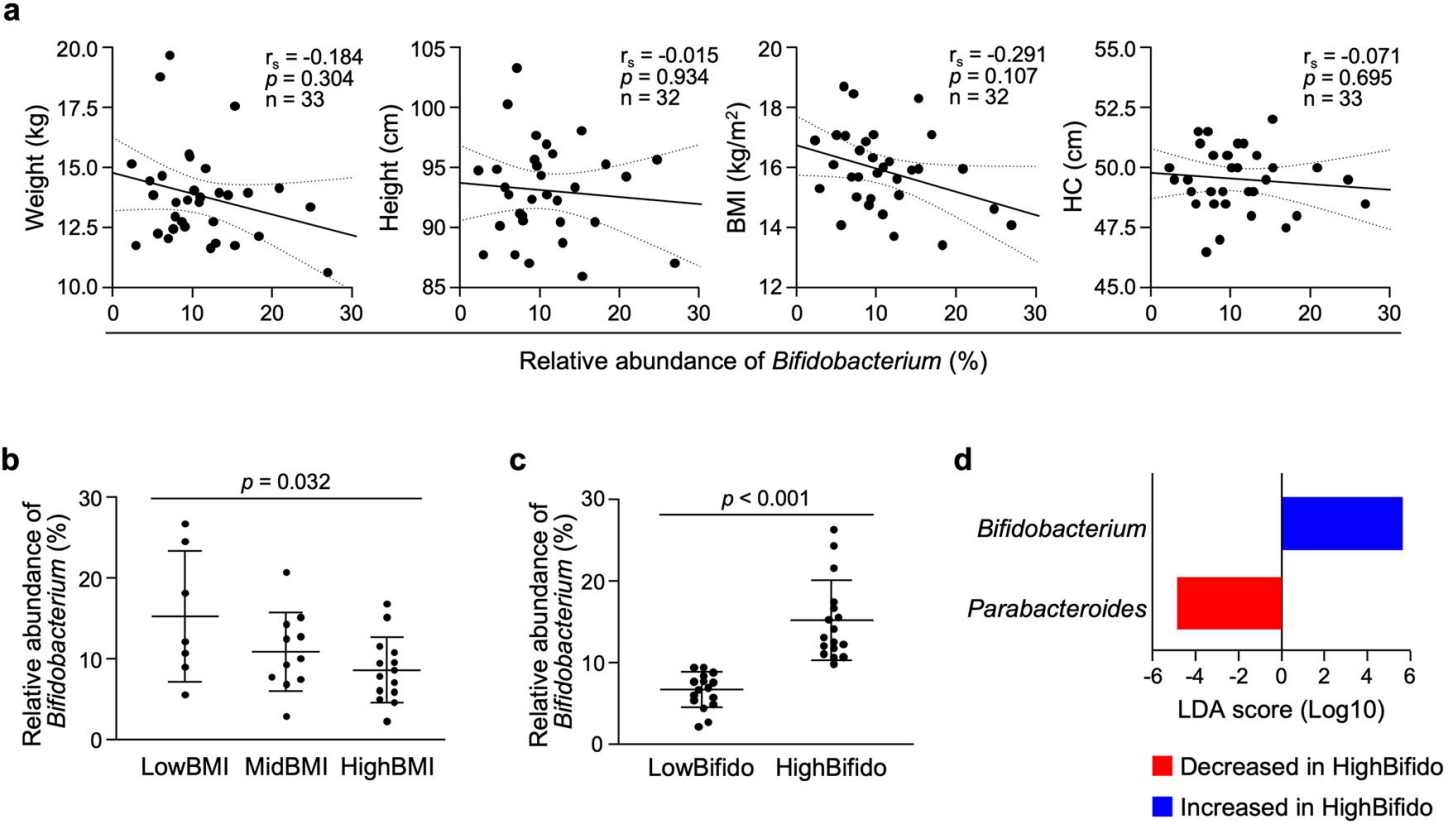
High relative abundance of *Bifidobacterium* in children at 3 y is associated with low BMI and compositional differences in the intestinal microbiota. **a** Correlation analysis between anthropological data and the relative abundance of *Bifidobacterium* in children at 3 y. **b** Comparison of the relative abundance of *Bifidobacterium* between the LowBMI (*n* = 7; BMI ≤ 33.3rd percentile in age- and sex-matched Japanese children), MidBMI (*n* = 11; 33.4–66.7th) and HighBMI (*n* = 14; >66.7th) groups in children at 3 y. **c** Comparison of the relative abundance of *Bifidobacterium* between the LowBifido (*n* = 16) and HighBifido (*n* = 17) groups, to which the children are assigned in ascending order of the relative abundance of *Bifidobacterium* at 3 y. **d** Differentially abundant genera between the LowBifido and HighBifido groups identified by LefSe analysis. The error bars represent the mean ± SD. Dashed lines in (**a**) represent the 95% confidence interval range. Statistical significance was evaluated by Spearman’s rank correlation coefficient test in (**a**), one-way ANOVA followed by Tukey’s multiple comparison test in (**b**), and the Mann-Whitney’s U test in (**c**). In (**d**), taxa with an |LDA score| > 2.0 were considered differentially abundant between the groups.

Then, the effects of the formation of the *Bifidobacterium*-rich microbiota on the physical development of children were analyzed. There was no statistically significant correlation between occupancy of *Bifidobacterium* genus and either weight, height, or head circumference, but there was a tendency (*p* = 0.107) for a negative correlation with BMI (Fig. 4a). To conduct a more detailed analysis, the participants were divided into LowBMI (*n* = 7; BMI percentile ≤ 33.3rd), MidBMI (*n* = 11; 33.4–66.7th percentile), and HighBMI (*n* = 14; >66.7th percentile) groups (Supplementary Table 3). As a result, the relative abundance of *Bifidobacterium* in the LowBMI group was significantly higher than that in the HighBMI group (Fig. 4b). Next, to clarify the effect of the high relative abundance of *Bifidobacterium* on the intestinal microbiota ecosystem, the children were divided into LowBifido (*n* = 16) and HighBifido (*n* = 17) groups in ascending order of the relative abundance of *Bifidobacterium* at 3 y, and the intestinal microbiota was compared between the groups. In the diversity analysis, the intestinal microbiota in the LowBifido and HighBifido groups showed significantly different composition in the β-diversity, whereas no difference was observed in the α-diversity (Supplementary Fig. 5a, b). In the genus-level analysis, a significant difference other than *Bifidobacterium* was only observed in the relative abundance of *Parabacteroides* between the groups, and *Parabacteroides* in the HighBifido group was lower than that in the LowBifido group (Fig. 4d, Supplementary Fig. 5c). These results indicate that the high relative abundance of *Bifidobacterium* in children at 3 y is associated with low BMI and the formation of the intestinal microbiota characterized by low *Parabacteroides*.

### High HD5 secretion is associated with a high relative abundance of *Bifidobacterium* in children during the early life, especially in the weaning period

**Fig. 5:**
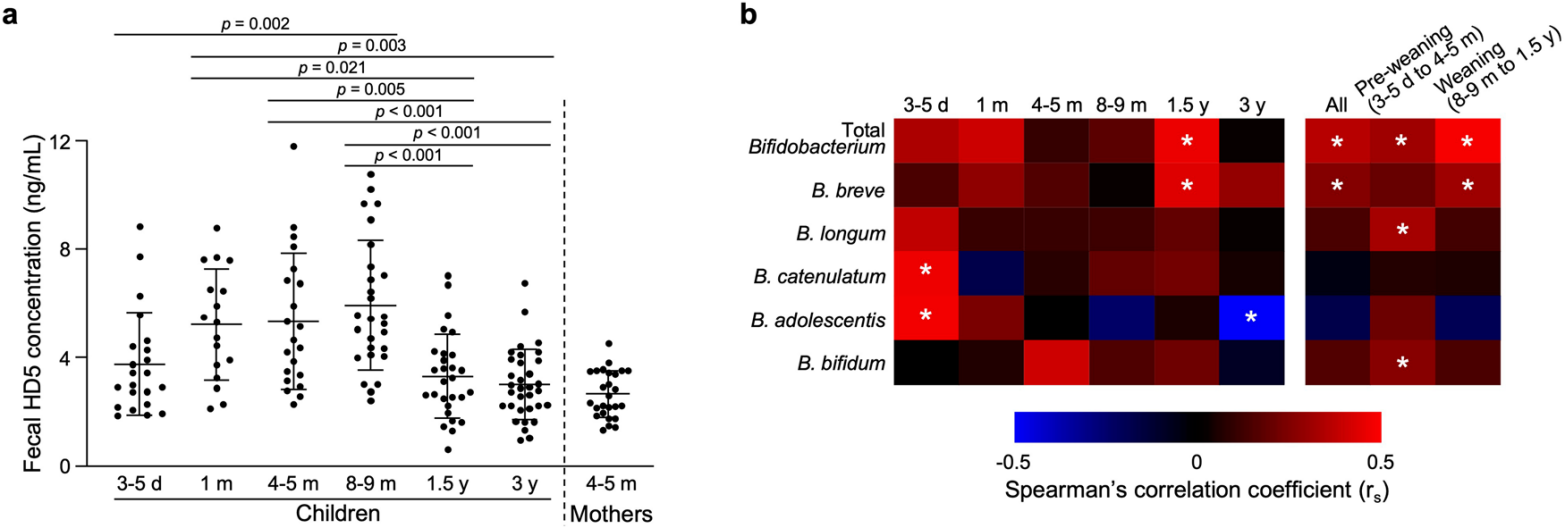
High HD5 concentration in feces is associated with a high relative abundance of *Bifidobacterium* of children in early life. **a** Transition of fecal HD5 concentration in children along with their development. **b** Correlation matrix between the relative abundance of each *Bifidobacterium species* and the fecal HD5 concentration in children at each age, all period (combined data set of all time points), the pre-weaning period (3–5 d, 1 m, and 4–5 m), and the weaning period. Error bars represent the means ± SD in (**a**). In (**b**), * indicates statistically significant correlation (*p* < 0.05). Statistical significance was evaluated by one-way ANOVA followed by Tukey’s multiple comparison test among children at each age in (**a**) and Spearman’s rank correlation coefficient test in (**b**).

**Fig. 6:**
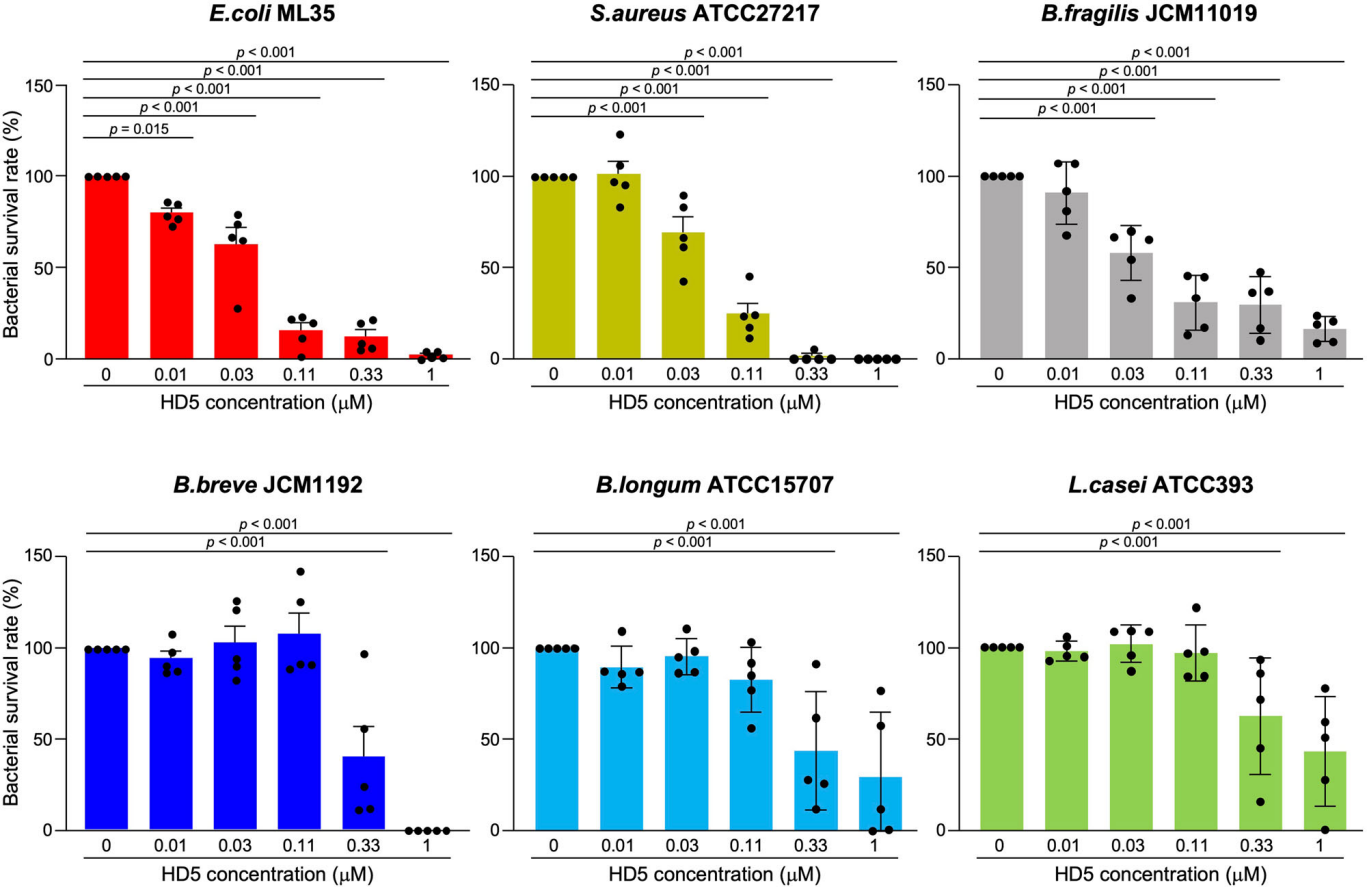
HD5 shows only minimal in vitro bactericidal activities against commensal bacteria at the sub-μM concentrations. In vitro bactericidal activities of HD5 against *Escherichia coli* (*E. coli*) ML35, *Staphylococcus aureus* (*S. aureus*) ATCC27217, *Bacteroides fragilis* (*B. fragilis*) JCM11019, *Bifidobacterium breve* (*B. breve*) JCM1192, *Bifidobacterium longum* (*B. longum*) ATCC15707, and *Lactobacillus casei* (*L. casei*) ATCC393. Five individual experiments were conducted, and the bacterial survival rate at each concentration was calculated relative to 0 μM concentration of each bacterium. The error bars represent the means ± SE. Statistical significance was evaluated by one-way ANOVA followed by Dunnett’s multiple comparison test against 0 μM.

Finally, the effect of HD5 secretion on the colonization of *Bifidobacterium* during early life was analyzed. HD5 was already detected in feces at 3–5 d, showed higher concentration than their mothers from 3–5 d to 8–9 m, after which it decreased to the same level as that in mothers after 1.5 y (Fig. 5a, Supplementary Table 4). In the correlation analysis between the fecal HD5 concentration and the relative abundance of total *Bifidobacterium* at each age, a positive correlation was observed at 1.5 y (Fig. 5b). In the analysis of individual *Bifidobacterium* species, fecal HD5 was positively correlated with *B. catenulatum* and *B. adolescentis* at 3–5 d and with *B. breve* at 1.5 y, and was negatively correlated with *B. adolescentis* at 3 y (Fig. 5b). In the analysis of each period integrating multiple age, HD5 at the all-time period (all age; all), pre-weaning period (3–5 d, 1 m, and 4–5 m; pre-weaning), and the weaning period (8–9 m and 1.5 y; weaning) were all positively correlated with the relative abundance of total *Bifidobacterium* (Fig. 5b, Supplementary Fig. 6). Among these time periods, the strongest positive correlation was observed at the weaning period (Spearman’s correlation coefficient *r*_s_ = 0.357 in all; 0.311 in pre-weaning; 0.484 in weaning), indicating that a high HD5 concentration is associated with a high relative abundance of *Bifidobacterium* throughout infancy, especially during weaning. No associations were found between mode of delivery and feeding and relative abundance of *Bifidobacterium* at 3 y (Supplementary Fig. 7). To clarify the effect of HD5 on the colonization of *Bifidobacterium* directly, we conducted in vitro bactericidal assay of HD5 against *B. breve* and *B. longum* focusing on the sub-μM concentration, which is approximately the estimated concentration of HD5 in the colonic lumen (Fig. 6)^66,67^. HD5 showed significant bactericidal activities against *E. coli* ML35, pathogenic *S. aureus* ATCC27217, and opportunistic *B. fragilis* JCM11019 at concentration higher than 0.01 μM, 0.03 μM, and 0.03 μM, respectively. In contrast, HD5 showed significant bactericidal activity against commensal *B. breve* JCM1192, *B. longum* ATCC15707, and *L. casei* ATCC393 at concentration higher than 0.33 μM. These results indicate that HD5 has weaker bactericidal activity against commensal bacteria including *Bifidobacterium* compared to non-commensal and opportunistic bacteria, suggesting that HD5 contributes to the colonization of *Bifidobacterium* in the intestine.

## Discussion

The appropriate development of the early-life intestinal microbiota is widely recognized as important for the establishment of healthy microbiota in later life as a part of the DOHaD theory^12^. Although several studies focused on specific periods or strains regarding later-life colonization of *Bifidobacterium* that appeared in the early-life intestine have been reported^31,68,69^, there are no comprehensive and longitudinal studies on the association of *Bifidobacterium* at each age in early life from neonatal to early childhood at either the community (genus)-level or species-level resolution. In this study, we revealed that the high relative abundance of *Bifidobacterium* at the weaning period around 1 y relating to HD5 secretion is associated with the establishment of a *Bifidobacterium*-rich microbiota in later life by using the longitudinal dataset obtained from children at 3–5 d to 3 y. Our results indicate that *Bifidobacterium* in early life may contribute to long-term health by supporting the establishment of future intestinal microbiota in addition to the well-known pathway by promoting the physiological development of host^15–19^, providing an expanded perspective on the role of early-life *Bifidobacterium* in DOHaD theory.

According to the species-level analysis, the relative abundance of *B. breve* at the weaning period was positively correlated with that of the total *Bifidobacterium* at 3 y, suggesting that *B. breve* has a central role in the establishment of *Bifidobacterium*-rich microbiota in later life. In addition, the positive interspecies correlation between *B. breve* at the weaning and *B. catenulatum* at 3 y indicates that individual *Bifidobacterium* species may indirectly contribute to the establishment of *Bifidobacterium*-rich microbiota in later life by supporting the growth of other *Bifidobacterium* species. In general, *Bifidobacterium* species have a high ability to assimilate glycans derived from the host (e.g., mucin, human milk oligosaccharide: HMO) and food (e.g., starch, maltodextrin) and support each other’s growth through the cross-feeding of mono- and disaccharides produced by glycan degradation^70^. Although *B. breve* has limited assimilation activity against HMOs compared with *B. bifidum* and *B. longum subsp. infantis*^71^, it can degrade a wide range of food-derived glycans to ulilize^70^. In addition, *B. breve* supports the growth of *B. bifidum* in the presence of food-derived glycans (starch and xylan)^72^. Therefore, *B. breve* at the weaning period may support the colonization of the overall *Bifidobacterium* community by cross-feeding glycan metabolites derived from food components. Future studies based on our findings are needed to reveal detailed interactions between *Bifidobacterium* species during the maturation process of the intestinal microbiota.

It has been reported that the copy number of *Bifidobacterium spp*. in feces is negatively correlated with BMI in children aged 3–11 y^73^. Because the clinical criteria for overweight and obesity in children based on BMI are ≥85th percentile and ≥95th percentile^74^, the finding of this study that the relative abundance of *Bifidobacterium* in the LowBMI group (bottom 1/3 percentiles) was higher than in the HighBMI group (top 1/3 percentiles) at 3 y does not directly indicate association between *Bifidobacterium* and childhood obesity. In addition, this study does not take into account the potential confounding between BMI and the intestinal microbiota caused by diet. However, given that children who exceed the 50th BMI percentile even once at regular checkups at 2, 3, and 4.5 y have a 4.5 to 8.2-fold higher risk of overweight at 12 y^75^, a high relative abundance of *Bifidobacterium* at 3 y may be associated with a reduced risk of obesity in later life. The finding that children with a high relative abundance of *Bifidobacterium* presented a low relative abundance of *Parabacteroides* in the intestinal microbiota, may be linked to previous studies reporting positive relationship between a high relative abundance of *Parabacteroides* and diseases such as atopic dermatitis and developmental disorders in children^76,77^. Taken together, these finding suggest that a high relative abundance of *Bifidobacterium* at 3 y contributes to a reduced future disease risk by promoting the appropriate development of children in association with the appropriate intestinal microbiota.

Although it has been reported that α-defensin is already expressed in the fetal human small intestine at embryonic weeks 13–16^51^, and that, in adults, the secretory amount of HD5 gradually decreases with aging^48^, the transition of HD5 secretion from the early postnatal period to infancy is completely unknown. In this study, we revealed that α-defensin secretion by human Paneth cells occurs vigorously from the neonatal period and revealed the maturation process of HD5 secretion to early childhood. These findings shed light on the role of innate enteric immunity in regulating the intestinal microbiota fulfilled by Paneth cell α-defensin in the early-life period, with a relatively limited immune system when adaptive immunity, with the exception of IgA, remains immature. Furthermore, although it has been reported that oral administration of HD5 to high-fat diet-fed mice normalizes lipid metabolism and glucose tolerance and increases the relative abundance of *Bifidobacterium*^78^, the effect of HD5 on *Bifidobacterium* in the human intestinal microbiota is not yet understood. We demonstrated that the secretory amount of HD5 was positively correlated with the relative abundance of *Bifidobacterium* in the early-life intestinal microbiota and that HD5 does not shows in vitro bactericidal activities against commensal *B. breve, B. longum*, and *L. casei* at concentration lower than 0.11 μM. The HD5 concentration in ileostomy fluids was reported to be 7.9 μg/mL (2.2 μM) in Crohn’s disease patients and 10.5 μg/mL (2.9 μM) in non-Crohn’s disease controls^66^; thus, HD5 concentration in ileal lumen is estimated to be 2–3 μM. In addition, it is reported that the concentration of Crp4, a Crp isoform, in the colonic lumen of ICR mice is ~1/50 of that in the ileal lumen^67^. Therefore, the HD5 concentration of the colonic lumen where *Bifidobacterium* colonizes is estimated to be ~0.05 μM. Taken together, these findings suggest that HD5 eliminates potentially harmful bacteria to the host whereas shows no bactericidal activities against *Bifidobacterium* at the physiological concentrations in the colon, suggesting that HD5 contributes to the colonization of *Bifidobacterium* in the human intestine and the establishment of healthy intestinal microbiota in early life. In contrast, in the correlation analysis between HD5 and individual *Bifidobacterium* species at each period, a positive correlation was observed with only *B. longum* and *B. bifidum* before weaning and with only *B. breve* in the weaning period. These results indicate that although HD5 is associated with *Bifidobacterium* colonization in the intestine, the relationship between HD5 and individual *Bifidobacterium* species flexibly changes with the developmental phase of the host, and is likely related to the intestinal environment with complex interactions among other intestinal bacteria, host immune factors, and environmental factors such as diet. Thus, further understanding of the regulatory mechanism of symbiosis with humans and *Bifidobacterium*, with a focus on HD5, remains a challenge to be addressed in future studies.

Recent studies revealed that supplementation with *Bifidobacterium* during the newborn to pre-weaning period contributes to children’s health by promoting the development of the immune system, bowel function, and weight gain^17,79^ and further reduces the risk of NEC^80^, suggesting the benefit of the administration of *Bifidobacterium* as probiotics during the pre-weaning period. Furthermore, the relative abundance of *Bifidobacterium* in adults inversely correlates with serum markers of chronic inflammation (CRP and IL-6)^25^, and adult patients with several diseases, such as type 2 diabetes and type B hepatitis, have lower *Bifidobacterium* abundance^24^. Additionally, high *Bifidobacterium* abundance is related to healthy aging and longevity^26,27^. Thus, the establishment of *Bifidobacterium*-rich microbiota not only in early life but also in adulthood is thought to be important for promoting lifelong health. However, a study on the oral administration of *B. longum* AH1206 to healthy adults revealed that AH1206 becomes undetectable in more than 70% of the participants within the first month after administration, and the colonization of AH1206 in the intestine after 6 months is found in fewer than 30% of the participants^81^, indicating that colonization of orally administered live bacteria as probiotics in adulthood is transient in most cases. In this study, we revealed that the colonization of *Bifidobacterium* in the weaning period correlates with the establishment of a *Bifidobacterium*-rich microbiota in later life, and implied that HD5 promotes the colonization of *Bifidobacterium* in this period and after. This study highlights the importance of the weaning period in promoting the establishment of healthy intestinal microbiota throughout life, indicating that the weaning period is a window of opportunity for intervention in the intestinal environment. Recent studies revealed that butyrate and leucine directly induce granule secretion from Paneth cells^42^ and several food factors, such as rutin, inulin, and arginine increase the number of Paneth cells in vivo and ex vivo^82–84^. Our findings further contribute to the development of an integrated strategy for improving the early-life intestinal environment that combines conventional pre/probiotics with certain dietary factors that activates Paneth cell function.

It should be noted that there are several limitations in this study, including a relatively small sample size and an observation period of 3 years in the longitudinal cohort. In addition, this study could not directly verify the effects of HD5 and *Bifidobacterium* in the weaning period on the future microbiota due to the basic nature of the observational study. Because the SMILE Iwamizama is an ongoing study, these limitations are expected to be addressed through validation studies using larger and longer-term datasets obtained from future follow-up and interventional trials targeting the weaning period. Despite these limitations, this study provides valuable insights into the mechanism for the establishment of *Bifidobacterium*-rich microbiota in humans relating to Paneth cell α-defensin and contributes to a better understanding of the interaction between humans and the intestinal microbiota in pursuit of life-course health from the early life to elderly.

## Supplementary information

Supplementary Information

Description of Additional Supplementary Files

Supplementary Data 1

Reporting Summary

## Data Availability

The 16S rRNA gene sequencing data associated with this study are publicly available in the NCBI BioProject PRJNA1265424. All numerical source data underlying the figures, tables, and supplementary items are also provided in the Supplementary Data 1. Metadata that could compromise the privacy of study participants are not publicly available except upon reasonable request to the corresponding author Kiminori N.

## Code availability

The microbiota bioinformatics was implemented in Qiime2 (ver. 2022.8), which is publicly available (https://qiime2.org). All the statistical analyses were conducted by using the commercially available GraphPad Prism version 9.0 software (GraphPad Software Inc., San Diego, CA). BMI and BMI percentile were calculated by using a Microsoft Excel-based tool for growth evaluation provided by the Japanese Society for Pediatric Endocrinology (http://jspe.umin.jp/medical/chart_dl.html). The codes used for the microbiota analysis that support the findings of this study are available upon reasonable request to the corresponding author Kiminori N.
