# Supplementary material for: Modulation of Bifidobacterium by HD5 during weaning is associated with high abundance in later life
Source: Commun Med (Lond). 2025 Jul 1;5:250. doi: 10.1038/s43856-025-00977-6 (PMC12219304; doi:10.1038/s43856-025-00977-6)
Supplement: Supplementary file 1 — Supplementary Information [file 43856_2025_977_MOESM1_ESM.pdf]

Supplementary Information File

Modulation of *Bifidobacterium* by HD5 during weaning is associated with high abundance in later life

Yu Shimizu, Yuki Yokoi, Shuya Ohira, Hirohisa Izumi, Satomi Kawakami, Miu Ihara, Fuka Tabata, Yasuhiro Takeda, Takashi Kimura, Koshi Nakamura, Akiko Tamakoshi, Tokiyoshi Ayabe, and Kiminori Nakamura

Supplementary Table 1. Summary of the maternal information, the mode of delivery and feeding of the children

**Maternal information**	
Age (years-old, mean ± S.D.)	31.4 ± 4.3
Early-pregnancy BMI (kg/m^2, mean ± S.D.)*1	21.6 ± 2.9
Gestational period (weeks, mean ± S.D.)*2	38.9 ± 1.0
**Delivery mode*3**	
Normal delivery	19
Caesarean section	4
**Feeding mode at each age**	
**(Exclusive breastfeeding/Partial or Exclusive Formula-feeding)**	
3-5 d*4	6/23
1 m*5	14/18
4-5 m*5	13/19

*1 Data was calculated based on the information obtained at the first clinical visit.

*2 Five participants lack the information.

*3 Ten participants lack the information.

*4 Four participants lack the information.

*5 One participant lacks the information.

Supplementary Table2. Summary of the fecal sample collection status for each child

	Mother	3-5 d	1 m	4-5 m	8-9 m	1.5 y	3 y
Child 1	√	√	√	N/A	√	√	√
Child 2	√	N/A	√	N/A	N/A	√	√
Child 3	N/A	N/A	N/A	N/A	N/A	√	√
Child 4	√	√	√	√	√	√	√
Child 5	N/A	√	√	√	√	√	√
Child 6	√	√	√	√	√	√	√
Child 7	√	√	√	√	N/A	N/A	√
Child 8	√	√	√	√	√	√	√
Child 9	√	√	√	√	√	√	√
Child 10	√	N/A	N/A	√	N/A	N/A	√
Child 11	√	√	N/A	N/A	√	√	√
Child 12	√	√	N/A	N/A	√	√	√
Child 13	√	√	N/A	N/A	√	N/A	√
Child 14	√	√	N/A	√	√	√	√
Child 15	√	N/A	N/A	N/A	N/A	√	√
Child 16	√	√	√	√	√	√	√
Child 17	√	√	N/A	√	√	√	√
Child 18	√	√	√	√	√	√	√
Child 19	√	N/A	N/A	N/A	√	√	√
Child 20	√	N/A	N/A	√	√	√	√
Child 21	√	√	N/A	√	√	√	√
Child 22	N/A	N/A	N/A	N/A	√	√	√
Child 23	√	N/A	N/A	√	√	√	√
Child 24	√	N/A	N/A	√	√	N/A	√
Child 25	√	√	√	√	√	√	√
Child 26	√	√	√	N/A	√	√	√
Child 27	N/A	√	√	N/A	√	√	√
Child 28	√	√	√	√	N/A	N/A	√
Child 29	√	√	√	√	√	√	√
Child 30	N/A	N/A	N/A	N/A	√	√	√
Child 31	√	√	√	√	√	√	√
Child 32	√	√	√	√	√	√	√
Child 33	√	N/A	N/A	√	√	√	√

√: Sample collection.

N/A: Not applicable

Supplementary Table 3. Characteristics of children in Low, Mid, and HighBMI group at 3 y

	LowBMI	MidBMI	HighBMI
Number of participants (male/female)	7 (4/3)	11 (8/3)	14 (10/4)
Range of BMI percentile	≤ 33.3rd	33.4 to 66.7th	> 66.7th
BMI (kg/m^2, Mean ± SD, Min–Max)	14.2 ± 0.5 (13.4–14.8)	15.6 ± 0.4 (15.0–16.0)	17.1 ± 0.9 (16.0–18.7)
Weight (kg, Mean ± SD, Min–Max)	12.4 ± 1.0 (10.7–13.6)	12.9 ± 0.9 (11.8–14.2)	15.3 ± 2.0 (12.8–19.7)
Height (cm, Mean ± SD, Min–Max)	93.3 ± 3.3 (87.1–97.0)	91.0 ± 3.2 (86.0–95.7)	94.6 ± 4.4 (87.1–103.3)
HC (cm, Mean ± SD, Min–Max)	48.9 ± 0.7 (48.0–50.0)	49.1 ± 1.1 (46.5–50.5)	50.0 ± 1.5 (47.0–52.0)

Supplementary Table 4. Comparison of fecal HD5 concentration between mothers and children at each timepoint

	HD5 concentration (ng/mL)	p value* (vs mothers at 4-5 m)
Mothers at 4-5 m	2.64 ± 0.85	-
Children at		
3-5 d	3.77 ± 1.89	0.030
1 m	5.23 ± 2.04	< 0.001
4-5 m	5.35 ± 2.51	< 0.001
8-9 m	5.93 ± 2.39	< 0.001
1.5 y	3.32 ± 1.56	0.083
3 y	3.01 ± 1.29	0.340

* Statistical significance was evaluated by Mann-Whitney's U-test.

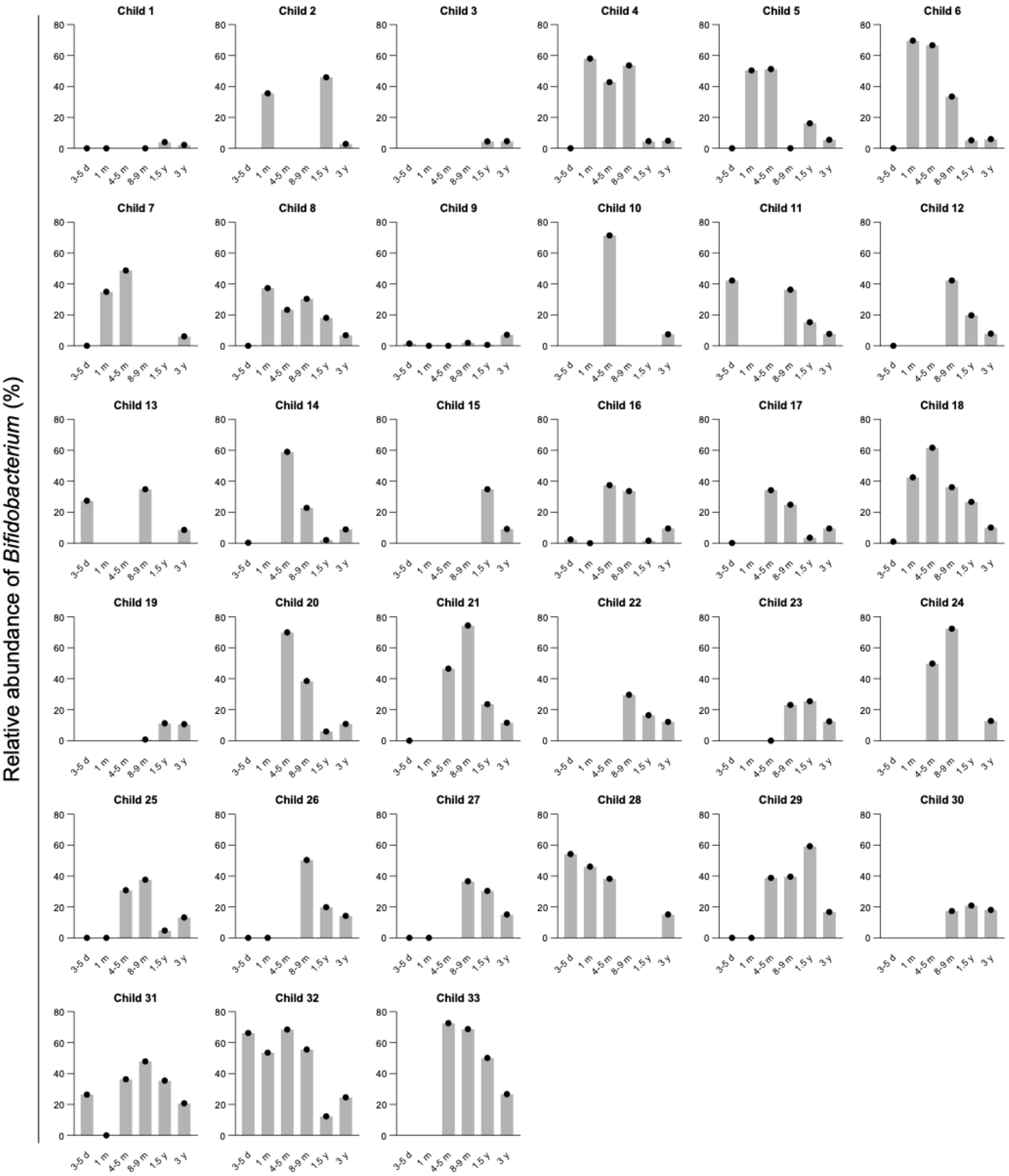

Supplementary Figure 1. Transition of Bifidobacterium occupancy in each child along with the development

Transition of relative abundance of *Bifidobacterium* genus in children along with their development. Children were arranged in ascending order of *Bifidobacterium* occupancy at 3 y.

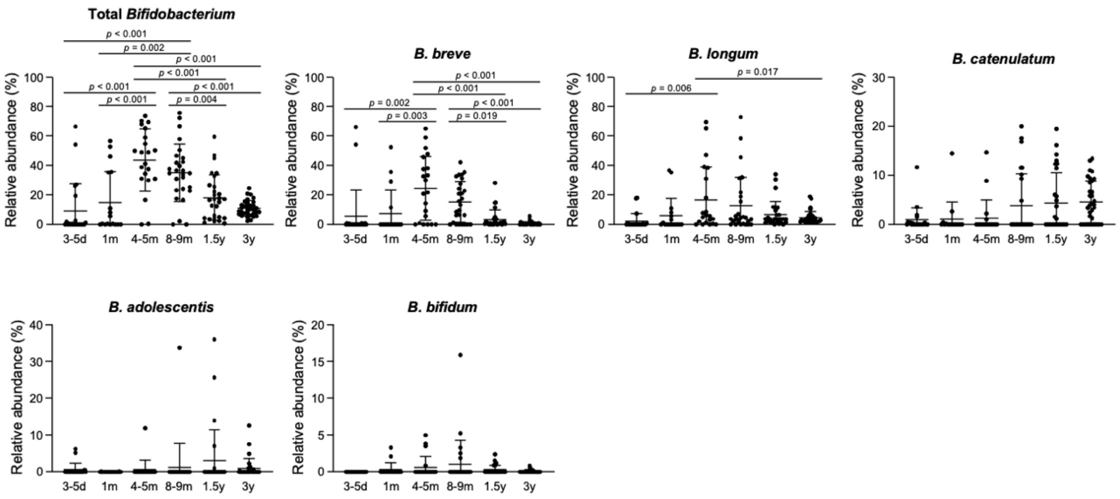

Supplementary Figure 2. Dot plots of relative abundance of each Bifidobacterium species in children

Transition of each *Bifidobacterium* species in children along with their development. Error bars represent mean ± S.D. Statistical significance was evaluated by one-way ANOVA followed by Tukey's multiple comparison test.

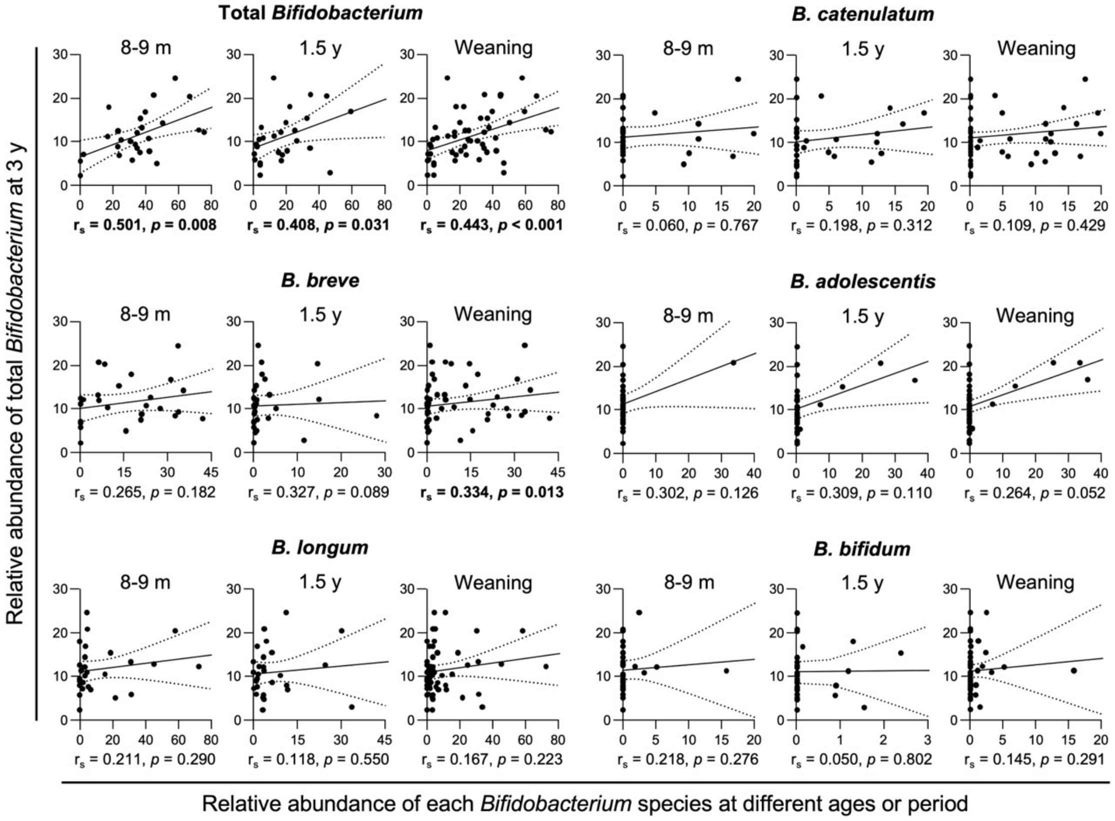

Supplementary Figure 3. Correlation analysis between the relative abundance of total Bifidobacterium of children at 3 y and each Bifidobacterium species at 8-9 m, 1.5 y, and the weaning period

Statistical significance was evaluated by Spearman's rank correlation coefficient test. Bold font in correlation coefficients (r_s) and *p* value means statistically significant ($p < 0.05$).

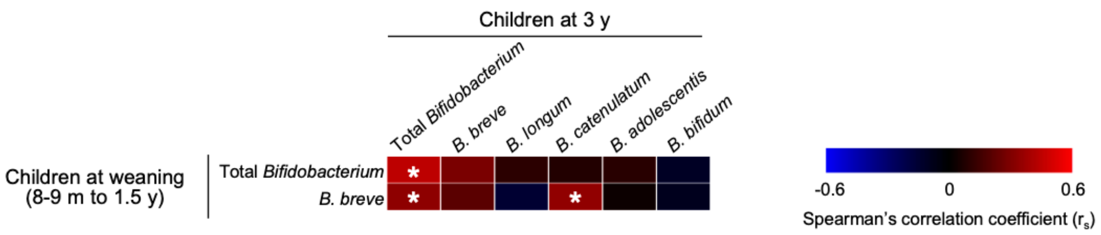

Supplementary Figure 4. Correlation matrix between each Bifidobacterium species in 3 y and total Bifidobacterium and B. breve in the weaning period

Statistical significance was evaluated by Spearman's rank correlation coefficient test. * in each cell means statistically significant correlation ($p < 0.05$).

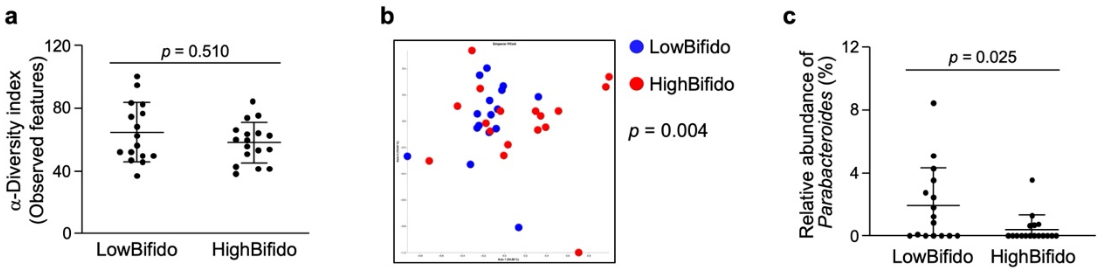

Supplementary Figure 5. Comparison of the intestinal microbiota between children of LowBifido and HighBifido group at 3 y

(a) Observed features, an index of α-diversity. (b) Principal coordinate plot based on weighted UniFrac distance, an index of β-diversity. (c) Relative abundance of *Parabacteroides*. Error bars represent mean ± S.D. Statistical significance was evaluated by Mann-Whitney's U-test in (a), (c) and PERMANOVA analysis in (b).

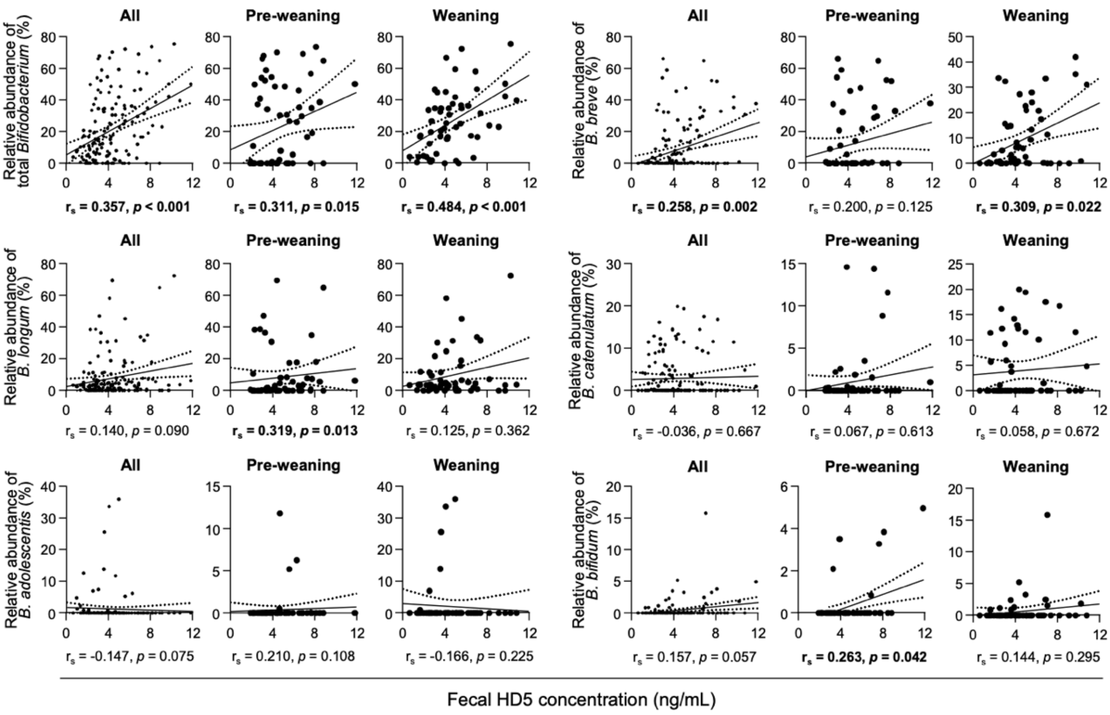

Supplementary Figure 6. Correlation analysis between fecal HD5 concentration and Bifidobacterium occupancy of children in all, pre-weaning, and weaning period

Correlation analysis between fecal HD5 concentration and relative abundance of each *Bifidobacterium* species of children in all, pre-weaning, and weaning period. Dashed lines represent the 95% confidence interval range. Statistical significance was evaluated by Spearman's rank correlation coefficient test. Bold font in correlation coefficients (r_s) and p value means statistically significant ($p < 0.05$).

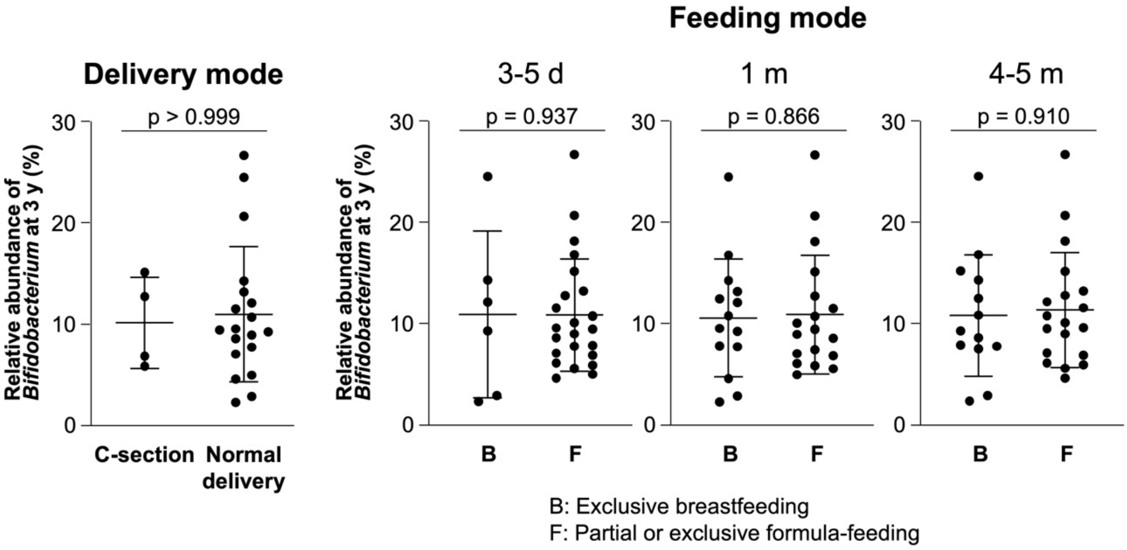

Supplementary Figure 7. Comparison of the relative abundance of *Bifidobacterium* at 3 y between different delivery or feeding mode groups.

Error bars represent mean ± S.D. Statistical significance was evaluated by Mann-Whitney's U-test.
