# Supplementary material for: Modulation of Bifidobacterium by HD5 during weaning is associated with high abundance in later life
Source: Commun Med (Lond). 2025 Jul 1;5:250. doi: 10.1038/s43856-025-00977-6 (PMC12219304; doi:10.1038/s43856-025-00977-6)
Supplement: Supplementary file 2 — Description of Additional Supplementary Files [file 43856_2025_977_MOESM2_ESM.pdf]

Description of Additional Supplementary Files

Supplementary Data 1

The numerical source data for all figures, tables, and supplementary items.
