# Supplementary material for: Modulation of Bifidobacterium by HD5 during weaning is associated with high abundance in later life
Source: Commun Med (Lond). 2025 Jul 1;5:250. doi: 10.1038/s43856-025-00977-6 (PMC12219304; doi:10.1038/s43856-025-00977-6)
Supplement: Supplementary file 4 — Reporting Summary [file 43856_2025_977_MOESM4_ESM.pdf]

nature portfolio

Corresponding author(s): Kiminori Nakamura

Last updated by author(s): May 19, 2025

Reporting Summary

Nature Portfolio wishes to improve the reproducibility of the work that we publish. This form provides structure for consistency and transparency in reporting. For further information on Nature Portfolio policies, see our Editorial Policies and the Editorial Policy Checklist.

Statistics

For all statistical analyses, confirm that the following items are present in the figure legend, table legend, main text, or Methods section.

n/a	Confirmed	
☐	☒	The exact sample size (n) for each experimental group/condition, given as a discrete number and unit of measurement
☐	☒	A statement on whether measurements were taken from distinct samples or whether the same sample was measured repeatedly
☐	☒	The statistical test(s) used AND whether they are one- or two-sided<br>*Only common tests should be described solely by name; describe more complex techniques in the Methods section.*
☐	☒	A description of all covariates tested
☐	☒	A description of any assumptions or corrections, such as tests of normality and adjustment for multiple comparisons
☐	☒	A full description of the statistical parameters including central tendency (e.g. means) or other basic estimates (e.g. regression coefficient) AND variation (e.g. standard deviation) or associated estimates of uncertainty (e.g. confidence intervals)
☐	☒	For null hypothesis testing, the test statistic (e.g. F, t, r) with confidence intervals, effect sizes, degrees of freedom and P value noted<br>*Give P values as exact values whenever suitable.*
☒	☐	For Bayesian analysis, information on the choice of priors and Markov chain Monte Carlo settings
☒	☐	For hierarchical and complex designs, identification of the appropriate level for tests and full reporting of outcomes
☐	☒	Estimates of effect sizes (e.g. Cohen's d, Pearson's r), indicating how they were calculated

Our web collection on statistics for biologists contains articles on many of the points above.

Software and code

Policy information about availability of computer code

Data collection	For data collection, no software was used in this study.
Data analysis	For Bacterial 16S rRNA gene-based taxonomic analysis, Qiime2 platform (ver. 2022.8) was used. For BMI and BMI-percentile calculation, Microsoft Excel-based tool for growth evaluation provided by the Japanese Society for Pediatric Endocrinology (http://jspe.umin.jp/medical/chart_dl.html) was used. For all statistical analyses, GraphPad Prism ver. 9.0 software (GraphPad Software Inc., San Diego, CA) was used.

For manuscripts utilizing custom algorithms or software that are central to the research but not yet described in published literature, software must be made available to editors and reviewers. We strongly encourage code deposition in a community repository (e.g. GitHub). See the Nature Portfolio guidelines for submitting code & software for further information.

Data

Policy information about availability of data

All manuscripts must include a data availability statement. This statement should provide the following information, where applicable:

- Accession codes, unique identifiers, or web links for publicly available datasets
- A description of any restrictions on data availability
- For clinical datasets or third party data, please ensure that the statement adheres to our policy

All numerical source data underlying the figures, tables, and supplementary items are provided in Supplementary Data 1. Other data are not publicly available due

to their containing information that could compromise the privacy of research participants, except upon direct request to the corresponding author, Kiminori N, with an appropriate research proposal.

Research involving human participants, their data, or biological material

Policy information about studies with human participants or human data. See also policy information about sex, gender (identity/presentation), and sexual orientation and race, ethnicity and racism.

Reporting on sex and gender	In this study, we did not collected gender data and collected sex data based on medical records. To focus on associations of the intestinal environment between mothers and their children, we included only female participants and their children (including both sex) in the analysis.
Reporting on race, ethnicity, or other socially relevant groupings	In our manuscript, we did not use socially constructed or socially relevant categorization variables such as race, ethnicity, gender identity, or socioeconomic status. The study population consisted of mothers and their children living in Iwamizawa City, Hokkaido, Japan, without using any criteria related to social constructs.<br>All participant data were obtained through self-report questionnaires and routine clinical assessments. There was no classification based on socially constructed categories.
Population characteristics	Participants in this study were mothers (31.4 ± 4.3 years-old) and their children living in Iwamizawa city, Hokkaido, Japan. Samples and anthropological data were longitudinally obtained from the children at postpartum 3-5 days, 1 month, 4-5 months, 8-9 months, 1.5 years, and 3 years.
Recruitment	Pregnant women living in Iwamizawa city are recruited when the municipal government issues the Maternal and Child Health Handbook and are enrolled in the cohort if written informed consent is obtained, and research consent from children was deemed based on consent signatures of their mothers.
Ethics oversight	This study was conducted in accordance with the guidelines laid down in the Declaration of Helsinki and all procedures involving human subjects were approved by the ethics committee of the Graduate School of Medicine at Hokkaido University and the Morinaga Milk Industry.

Note that full information on the approval of the study protocol must also be provided in the manuscript.

Field-specific reporting

Please select the one below that is the best fit for your research. If you are not sure, read the appropriate sections before making your selection.

☒ Life sciences ☐ Behavioural & social sciences ☐ Ecological, evolutionary & environmental sciences

For a reference copy of the document with all sections, see nature.com/documents/nr-reporting-summary-flat.pdf

Life sciences study design

All studies must disclose on these points even when the disclosure is negative.

Sample size	No sample size calculation was performed in this study.
Data exclusions	In bacterial 16S rRNA gene-based taxonomic analysis, samples that did not meet the criteria for the quality and quantity of the sequencing data were excluded from the analysis.
Replication	Due to the specific features of the dataset used in this study, we have not assessed the replication of our findings.
Randomization	Because the main purpose of this study is not to compare between groups but to elucidate the transition of the intestinal environment during child development, randomized allocation of participants is not relevant to this study.
Blinding	Because the main purpose of this study is not to compare between groups but to elucidate the transition of the intestinal environment during child development, blinding of experimental group is not relevant to this study.

Reporting for specific materials, systems and methods

We require information from authors about some types of materials, experimental systems and methods used in many studies. Here, indicate whether each material, system or method listed is relevant to your study. If you are not sure if a list item applies to your research, read the appropriate section before selecting a response.

Materials & experimental systems

n/a	Involved in the study
☐	☒ Antibodies
☒	☐ Eukaryotic cell lines
☒	☐ Palaeontology and archaeology
☒	☐ Animals and other organisms
☐	☒ Clinical data
☒	☐ Dual use research of concern
☒	☐ Plants

Methods

n/a	Involved in the study
☒	☐ ChIP-seq
☒	☐ Flow cytometry
☒	☐ MRI-based neuroimaging

Antibodies

Antibodies used

For quantification of fecal HD5 concentration, monoclonal antibodies against HD5 and sandwich ELISA system established in our previous study was used.

Validation

Establishment process of the antibodies and sandwich ELISA was described in our previous study (doi: 10.1007/s11357-021-00398-y).

Clinical data

Policy information about clinical studies

All manuscripts should comply with the ICMJE guidelines for publication of clinical research and a completed CONSORT checklist must be included with all submissions.

Clinical trial registration

This study was approved and registered by the ethics committees of the Graduate School of Medicine, Hokkaido University (approval ID: 16-039) and Morinaga Milk Industry Co., Ltd. (16005-144).

Study protocol

Pregnant women and their children were enrolled in the study. Fecal samples were obtained from both from mothers and children. A small part of each fecal sample is collected by brush-type collection kits containing guanidine thiocyanate for the intestinal microbiota analysis. The remaining fecal samples are stored at -80? until use and are subjected to a series of analyses including HD5 quantification. In addition, anthropological data of the children measured at the time of regular check-ups is provided by the mothers at each time point.

Data collection

Mothers and children living in Iwamizawa city, Hokkaido, Japan were recruited. Fecal samples are collected from children at postpartum 3-5 days, 1 months, 4-5 months, 8-9 months, 1.5 years, 3 years, and from mothers at 4-5 months. In addition, anthropological data of the children measured at the time of regular check-ups is provided by the mothers at each time point.

Outcomes

The outcome of this study is longitudinal association between the intestinal microbiota and HD5 secretion during child development. The intestinal microbiota was assessed by bacterial 16S ribosomal RNA gene sequencing using bacterial DNA extracted from feces, and HD5 secretion was assessed by quantification of HD5 concentration in fecal extracts by sandwich ELISA.

Plants

Seed stocks

N/A

Novel plant genotypes

N/A

Authentication

N/A
